# Supplementary material for: Participant engagement and feedback in microbiome projects: a case of AWI-Gen 2
Source: mSystems. 2026 Jul 28;11(8):e01800-25. doi: 10.1128/msystems.01800-25 (PMC13483426; doi:10.1128/msystems.01800-25)
Supplement: Supplementary file 3 [file reviewer-comments.pdf]

AMERICAN SOCIETY FOR MICROBIOLOGY | mSystems®

Participant engagement and feedback in microbiome projects: a case of AWI-Gen 2

Claudine Nkera-Gutabara, Luicer Olubayo, Ovokeraye Oduaran, Isaac Kisiangani, Simon Khoza, Khulu Gama, Meriam Maritze, Carol Mabunda, Dorcas Keya, Kayode Adetunji, Stephen Tollman, Lisa Micklesfield, Shukri Mohamed, F. Gómez-Olivé Casas, Furahini Tluway, Michèle Ramsay, Ami Bhatt, Scott Hazelhurst, and Dylan Maghini

Corresponding Author(s): Luicer Olubayo, University of the Witwatersrand Johannesburg Sydney Brenner Institute for Molecular Bioscience

Review Timeline:

Submission Date:	December 31, 2025
Editorial Decision:	April 10, 2026
Revision Received:	April 30, 2026
Accepted:	June 15, 2026

Editor: Amber Benezra

Reviewer(s): Disclosure of reviewer identity is with reference to reviewer comments included in decision letter(s). The following individuals involved in review of your submission have agreed to reveal their identity: Kristien Nel Van Zyl (Reviewer #1)

Transaction Report:

(Note: With the exception of the correction of typographical or spelling errors that could be a source of ambiguity, letters and reports are not edited. The original formatting of letters and referee reports may not be reflected in this compilation.)

DOI: https://doi.org/10.1128/msystems.01800-25

Re: mSystems01800-25 (Participant engagement and feedback in microbiome projects: a case of AWI-Gen 2)

Dear Dr. Luicer Anne Ingasia Olubayo:

Thank you for the privilege of reviewing your work. Below you will find instructions from the mSystems editorial office, and the reviewer comments. Minor modifications are requested: specifically Reviewer #2's request for more details about the study population, and clarification of some clinical aspects of the study.

Please return the manuscript within 60 days; if you cannot complete the modification within this time period, please contact me. If you do not wish to modify the manuscript and prefer to submit it to another journal, notify me immediately so that the manuscript may be formally withdrawn from consideration by mSystems.

Revision Guidelines
To submit your modified manuscript, log into the submission site at https://msystems.msubmit.net/cgi-bin/main.plex. Go to Author Tasks and click the appropriate manuscript title to begin. The information you entered when you first submitted the paper will be displayed; update this as necessary. Note the following requirements:

• Upload point-by-point responses to the issues raised by the reviewers in a file named "Response to Reviewers," NOT in your cover letter.
• Upload a compare copy of the manuscript (without figures) as a "Marked-Up Manuscript" file.
• Upload a clean .DOC/.DOCX version of the revised manuscript and remove the previous version.
• Each figure must be uploaded as a separate, editable, high-resolution file (TIFF or EPS preferred), and any multipanel figures must be assembled into one file.
• Any supplemental material intended for posting by ASM should be uploaded with their legends separate from the main manuscript. You can combine all supplemental material into one file (preferred) or split it into a maximum of 10 files with all associated legends included.

For complete guidelines on revision requirements, see our Submission and Review Process webpage. Submission of a paper that does not conform to guidelines may delay acceptance of your manuscript.

Data availability: ASM policy requires that data be available to the public upon online posting of the article, so please verify all links to sequence records, if present, and make sure that each number retrieves the full record of the data. If a new accession number is not linked or a link is broken, provide mSystems production staff with the correct URL for the record. If the accession numbers for new data are not publicly accessible before the expected online posting of the article, publication may be delayed; please contact production staff (mSystems@asmusa.org) immediately with the expected release date.

Publication Fees: For information on publication fees and which article types are subject to charges, visit our website. If your manuscript is accepted for publication and any fees apply, you will be contacted separately about payment during the production process; please follow the instructions in that e-mail. Arrangements for payment must be made before your article is published.

ASM Membership: Corresponding authors may join or renew ASM membership to obtain discounts on publication fees. Need to upgrade your membership level? Please contact Customer Service at Service@asmusa.org.

The ASM Journals program strives for constant improvement in our submission and publication process. Please tell us how we can improve your experience by taking this quick Author Survey.

Thank you for submitting your paper to mSystems.

Sincerely,
Dr. Amber Benezra
Editor
mSystems

Reviewer #1 (Comments for the Author):

I applaud the authors for including this sub-study in their research. The need for community-involved and -driven research in Africa has become increasingly recognized. It should be a requirement for any future studies to include some form of participant or community engagement.

Reviewer #2 (Comments for the Author):

The work addresses a critical gap in microbiome research ethics and implementation, particularly in underrepresented populations.

Strengths
1. High Novelty and Relevance
Returning individualized microbiome results in low-resource settings is underexplored, and this study provides rare empirical data.
2.Strong contribution to ethical microbiome research and global health equity.

Major Concerns
1. Limited Generalizability
Study population: women aged 42-86 only
This significantly limits applicability across:
Younger populations
Men
Different sociocultural groups

Explicitly discuss how gender/age may influence:
comprehension
engagement
behavioral response

Microbiome Report Content Not Fully Described
It is unclear:
What exactly participants received
Level of personalization
Clinical vs descriptive interpretation

Response to reviewers

Reviewer #1

We thank the reviewer for the positive and encouraging assessment of our work. We appreciate the recognition of the importance of community-involved and community-driven research in African settings. We share the view that participant and community engagement should be an integral component of future studies, particularly where biological samples and complex data are involved. We are grateful for this supportive comment.

Reviewer #2

We thank the reviewer for the thoughtful and constructive comments and for recognizing the novelty and ethical importance of this work. We have revised the manuscript to address the concerns raised.

1. Limited generalizability / study population

We now clarify that the engagement study reflects the demographic composition of the parent AWI-Gen 2 microbiome sub-study, which sampled women aged 42–86 years, rather than an additional restriction introduced for the qualitative component. We also added discussion of how age, gendered health roles, and generational differences in education and health experience may shape comprehension, engagement, and behavioural response to microbiome feedback. These issues are now addressed in the "Participant selection" section (lines 195-198), the "Discussion" section (lines 511-520; 600-608), the "Limitations" section (lines 614-617), and the "Abstract" (line 50-51).

2. Microbiome report content and level of personalization

We have expanded the Methods section by adding a dedicated subsection, "Description of individualised microbiome reports"- lines 219-235, to clarify what participants received. We now specify that reports included personalised but descriptive/comparative information on microbial diversity and selected compositional features in relation to the broader AWI-Gen microbiome study population, rather than against a clinical reference standard. We also clarify that the reports were framed as educational rather than clinical and did not provide diagnoses, disease-risk prediction, treatment recommendations, or individualised medical advice.

3. Clinical versus descriptive interpretation

Relatedly, we now clarify throughout the manuscript that the returned microbiome results were non-diagnostic and were communicated with explicit discussion of uncertainty and current interpretive limits. We also emphasize the value of interactive feedback formats for explaining uncertainty and addressing participant questions in real time.

Re: mSystems01800-25R1 (Participant engagement and feedback in microbiome projects: a case of AWI-Gen 2)

Dear Dr. Luicer Anne Ingasia Olubayo:

The authors have addressed reviewer comments, and the additional context is helpful. We are pleased to accept your manuscript for publication. The only thing we would like to see added is a Data Availability Statement, as the heading is present but no content provided. Please indicate if any interview data would be made available upon request, and include a statement about where to find the microbiome data which is referenced should be included, even if the location of the microbiome data is referenced elsewhere in the text.

Your manuscript has been accepted, and I am forwarding it to the ASM production staff for publication. Your paper will first be checked to make sure all elements meet the technical requirements. ASM staff will contact you if anything needs to be revised before copyediting and production can begin. Otherwise, you will be notified when your proofs are ready to be viewed.

Data Availability: ASM policy requires that data be available to the public upon online posting of the article, so please verify all links to sequence records, if present, and make sure that each number retrieves the full record of the data. If a new accession number is not linked or a link is broken, provide production staff with the correct URL for the record. If the accession numbers for new data are not publicly accessible before the expected online posting of the article, publication may be delayed; please contact ASM production staff immediately with the expected release date.

Publication Fees: For information on publication fees and which article types have charges, please visit our website. We have partnered with Copyright Clearance Center (CCC) to collect author charges. If fees apply to your paper, you will receive a message from no-reply@copyright.com with further instructions. For questions related to paying charges through RightsLink, please contact CCC at ASM_Support@copyright.com or toll free at +1-877-622-5543. CCC makes every attempt to respond to all emails within 24 hours.

ASM Membership: Corresponding authors may join or renew ASM membership to obtain discounts on publication fees. Need to upgrade your membership level? Please contact Customer Service at Service@asmusa.org.

Embargo Policy: A press release may be issued as soon as the manuscript is posted on the mSystems Latest Articles webpage. The corresponding author will receive an email with the subject line "ASM Journals Author Services Notification" when the article is available online.

Cover Image Submissions: If you would like to submit a potential Cover Image, please email a file and a short legend to msystems@asmusa.org. Please note that we can only consider images that (i) the authors created or own and (ii) have not been previously published. By submitting, you agree that the image can be used under the same terms as the published article. Image File requirements: TIF/EPS, 7.5 inches wide by 8.25 inches tall (at least 2,250 pixels wide by 2,475 pixels tall), minimum 300 dpi resolution (600 dpi preferred), RGB, and no figure elements, e.g., arrows or panel labels. The legend should be a short description of the image, 1-2 sentences recommended. Please download and use this interactive template in Adobe to ensure that your proposed cover image meets our size requirements (https://journals.asm.org/pb-assets/pdf-text-excel-files/ASM-Interactive-Sizing-Cover-Template-1715689791.pdf).

Thank you for submitting your paper to mSystems.

Sincerely,
Sue Ishaq
Senior Editor
mSystems
